# Energy-efficient communication between IoMT devices and emergency vehicles for improved patient care

**DOI:** 10.1371/journal.pone.0330695

**Published:** 2025-08-28

**Authors:** Radwa Ahmed Osman

**Affiliations:** Basic and Applied Science Institute, College of Engineering, Arab Academy for Science, Technology and Maritime Transport, Alexandria, Egypt; Najran University College of Computer Science and Information Systems, SAUDI ARABIA

## Abstract

The rising integration of emergency healthcare services with the Internet of Medical Things (IoMT) creates a significant opportunity to improve real-time communication between patients and emergency vehicles like ambulances. Fast and reliable data interchange is crucial in an emergency, especially for those with chronic conditions who rely on wearable IoMT devices to monitor vital health signs. However, establishing consistent communication in real-world conditions such as restricted signal strength, changing distances, and power constraints remains a major difficulty. This paper provides an intelligent communication framework that uses a one-dimensional deep convolutional neural network (1D-CNN) and Lagrange optimization techniques to improve energy efficiency and data transmission speeds. Unlike many earlier models, our technique takes into consideration real-world characteristics such as signal-to-interference-plus-noise ratio (SINR), transmission power, and the distance between the ambulance and the patient’s device. The primary goal is to identify the ideal communication distance for dependable, energy-efficient data transfer during urgent emergency situations. The findings show that the suggested system enhances communication reliability, consumes less energy, and increases the possible data rate. This framework accelerates, smartens, and strengthens emergency healthcare communication systems by combining deep learning and mathematical optimization. These findings contribute to the progress of intelligent healthcare infrastructure, opening the way for responsive and dependable emergency services that can adapt to changing conditions while maintaining high performance and patient safety.

## 1 Introduction

The Internet of Things (IoT) has profoundly impacted a variety of industries, including agriculture [1,2] and healthcare. Its ability to join devices and provide smooth data flow has made it an essential technology in current medical systems. The Internet of Medical Things (IoMT), a subset of IoT, has been widely recognized for its potential to relieve burden on healthcare infrastructures by enabling remote monitoring, continuous patient evaluation, and real-time data analysis [3,4]. Recent technological breakthroughs in artificial intelligence (AI), 5G and Beyond 5G (B5G) communication systems, blockchain, and wearable sensors have hastened the integration of IoT into medical applications. These improvements have improved the reliability, efficiency, and scalability of smart healthcare systems [5,6]. Unlike traditional systems, these networked environments enable autonomous data flow between devices, decreasing the need for manual input and boosting decision-making processes. Wireless Sensor Networks (WSNs), in particular, serve an important role in continually monitoring crucial health indicators including heart rate, glucose levels, breathing patterns, and body temperature. Real-time transmission of data to healthcare facilities enables prompt treatments, efficient resource allocation, and better patient outcomes [7].

Despite the significant benefits of IoT in healthcare, a number of crucial difficulties remain—particularly when IoT devices must connect with emergency vehicles. Network problems include node and connection failures, latency concerns, security and privacy vulnerabilities, inadequate communication protocols, and general network inefficiencies [8,9]. Reliable and low-latency communication is crucial for transmitting time-sensitive data from wearable IoMT devices to moving ambulances or control centers during emergencies [10]. The COVID-19 pandemic highlighted the need for reliable IoT infrastructure, with research examining how IoT technology improved patient tracking, diagnostics, and remote care [?]. Poor communication quality during crises can have a direct impact on patient outcomes, especially when real-time data is lost, delayed, or damaged during transmission [11]. While the literature has extensively addressed security, privacy, and interoperability in IoT systems [12], less attention has been paid to performance-degrading factors like signal interference, variable SINR conditions, and transmission distance constraints—especially in dynamic real-world settings such as vehicle-to-device communication. To address these difficulties, academics are integrating AI, ML, and blockchain technologies into IoT ecosystems [13]. 5G and B5G networks provide high bandwidth and low latency, making them perfect for emergency communication with mobile devices and vehicles [14,15]. AI-driven optimization approaches can enhance communication system flexibility and robustness by optimizing crucial parameters in real-time constraints [16,17].

In dynamic healthcare communication contexts, D2D, V2V, and CUE connections frequently share the 5G spectrum alongside IoT and IoMT devices. This spectrum sharing causes interference on the receiver side, which can drastically reduce the reliability and performance of IoT systems by causing data corruption, increased delay, or entire loss of important health information. To address these issues, this paper introduces a new communication model that combines a one-dimensional Convolutional Neural Network (1D-CNN) with Lagrange optimization to improve the reliability and efficiency of data transmission between wearable IoT devices and rescue vehicles. The model is especially developed to take into consideration real-world physical restrictions like as interference levels, signal-to-noise ratio (SINR), transmission power, and device distance. The suggested technique preserves the integrity and availability of critical patient data by learning complicated patterns in communication quality and improving essential transmission parameters, resulting in more reliable emergency medical responses for people with chronic conditions. The following are this paper’s main contributions:

- A new communication architecture is presented to increase data transmission reliability between emergency vehicles and IoT devices used by patients with chronic conditions, while also lowering interference from other devices running in the same frequency range.
  A 1D-Convolutional Neural Network (1D-CNN) is combined with Lagrange optimization to address a communication reliability problem with realistic network restrictions.
- The proposed model optimizes the transmission distance between IoT devices and ambulances by taking into account major physical characteristics such path loss, SINR threshold $\left({\text{SINR}}_{th}\right)$, transmission power, and interference distance.
- The optimization approach takes into account interference from both IoT and emergency vehicle communications to anticipate the maximum permitted transmission range while minimizing data loss.
- The suggested system’s performance is assessed in terms of energy efficiency and feasible data rate under varied channel parameters, such as SINR, power control (PC), and distance, providing practical insights into IoT network design for healthcare situations.

The paper is organized as follows: An overview of relevant IoT research is given in Sect 2. The suggested methodology is described in depth in Sect 3. The approach’s analytical and experimental validation is covered in Sect 4. Finally, Sect 5 concludes the paper by outlining prospective future research directions.

## 2 Related work

The Internet of Things (IoT) improves healthcare efficiency by enabling real-time gathering, analysis, and transmission of patient data across medical networks [18]. To solve communication issues in autonomous systems, an adaptive AV2X model was suggested in [19], merging distributed deep learning with Lagrange optimization to find appropriate inter-vehicle distances. Under varied channel circumstances, this model beat standard techniques in terms of data throughput and energy efficiency. In healthcare, [20] introduced a smart health analytics solution for IoT networks that uses machine learning to manage massive amounts of medical data. Their strategy relied on mobile agents and clever protocols to assure quality-aware data services, reduce communication overhead, and improve data security via cryptographic techniques. The suggested solution outperformed previous methods in terms of network performance measures. In [21], a distributed deep learning architecture with Lagrange optimization was used to track COVID-19. By optimizing RFID reader location, the system increased data dependability and established the appropriate transmission distance for effective health monitoring. In addition, [22] suggested an energy-efficient healthcare monitoring method for the Internet of Medical Things (IoMT). The technique addressed latency and power consumption by employing fuzzy logic for data categorization and fog-based grouping depending on CPU performance and energy levels. This solution provided up to 77% energy savings and significantly decreased end-to-end latency across several QoS criteria when compared to conventional schemes.

The study in [23] presented an energy-optimized massive multiple-input multiple-output (MIMO)-enabled non-orthogonal multiple access (NOMA) IoT network for post-5G communication systems, aiming for ultra-fast data transfer and hyperconnectivity. This solution used large MIMO systems’ scalability to address issues such as imprecise channel state information and increased power consumption. To improve power allocation, the authors used fractional programming and successive convex approximation, which outperformed established approaches in terms of convergence time, energy efficiency, and user fairness. [24] used Deep Belief Networks (DBN) to optimize resource allocation in 5G-enabled IoT communications. The DBN architecture prioritized key IoT devices and achieved a 74% job completion rate, surpassing traditional allocation techniques. The Elliptic Curve Cryptography-based Energy-Efficient Routing Protocol (ECC-EERP) was developed by [25] to combine energy efficiency and security protocols. This protocol provided a safe and power-efficient routing method specifically designed for healthcare IoT systems. Additionally, [26] investigated energy-efficient scheduling in cloud-based smart city data centers for IoT workloads. Their approach offered an improved Poisson task model, as well as a predictive and adaptive energy management strategy, which outperformed current scheduling algorithms.

In addition, [27] proposed an IoT-based remote health surveillance system for isolated COVID-19 patients in rural places. The system used local sensors to monitor vital data including ECG, SpO, body temperature, and pulse rate. In an emergency, it sent GSM messaging, position tracking, and email warnings to physicians, while an AI-powered auto-injection system provided dosages in the absence of a healthcare practitioner, improving healthcare access through real-time monitoring and intervention. Similarly, [28] presented a drug monitoring architecture utilizing IoT and deep learning to ensure precise medication tracking during the COVID-19 epidemic. This method reduced human error, allowed for remote monitoring, and provided physicians with fast drug status information. The experimental assessment revealed better monitoring accuracy and treatment dependability. [29] proposed an IoT-based spatial health planning method using big data and visual analytics to address urban public health planning. Their methodology streamlined geographical data collecting and facilitated efficient public health evaluations, facilitating the complete design and allocation of urban healthcare resources. In line with healthcare digitalization, [30] developed a digital health management system that utilizes IoT and IoMT devices to monitor multimodal signals. The method centered on lower-limb rehabilitation, including wearable monitoring devices to increase diagnostic accuracy and therapeutic tailoring. Finally, during civil unrest, [31] created a deep learning-based IoT platform for real-time categorization of protest occurrences. This technology allowed for the intelligent distribution of healthcare resources by distinguishing protest kinds and intensities, with promising results for real-world implementation in mass emergency settings.

Despite significant advances in IoT-based healthcare systems, there is still a key gap in guaranteeing consistent communication between emergency response vehicles and patients with chronic diseases, particularly in real-world interference scenarios. This work tackles this important issue by utilizing the Internet of Things (IoT) to create robust, trustworthy, and interference-resistant communication between mobile emergency responders and wearable medical equipment. A primary goal is to establish the ideal communication distance that maximizes data dependability and energy economy, even when numerous devices are operating in the same frequency band. To do this, the proposed approach combines a distributed deep learning model and Lagrange-based analytical optimization. The deep learning component, based on a one-dimensional convolutional neural network (1D-CNN), learns patterns of signal deterioration and interference, allowing IoT devices to dynamically change their transmission behavior. Simultaneously, the Lagrange optimization approach discovers the ideal operational parameters, such as SINR, power, and distance, to ensure reliable communication while consuming the least amount of energy. The framework’s efficacy is measured using two major metrics: energy efficiency and feasible data rate. By combining these indications, the suggested method considerably increases the reliability and responsiveness of IoT communication in emergency healthcare situations. Finally, our study advances IoT-enabled health infrastructure by facilitating smooth, fast, and robust data transmission during life-critical circumstances. The constraints highlighted in the preceding investigations drive our suggested paradigm, which is described in the next section.

## 3 Proposed model

This section uses an analytical optimization strategy to present the suggested method for increasing communication between emergency vehicles and IoT devices used by chronic patients. The dataset obtained from the analytically proposed model is then validated using a deep neural network architecture that may be applied to real-world IoT networks. The goal of the analytical optimization approach is to pinpoint the crucial elements that guarantee dependable and energy-efficient data transfer, such as ideal communication distances and interference control methods. This entails simulating a number of real-world situations, such as the existence of interference from other devices using the same frequency band, and figuring out ways to reduce interruptions while preserving high data rates.

This framework is improved by the proven deep neural network architecture, which learns from the analytically generated data to forecast the best configurations in dynamic contexts. The model is made to adjust in real time to changes in environmental conditions, patient mobility, and the density of transmitting devices. This guarantees that the IoT devices can dynamically modify their configurations to get optimal communication efficiency. The suggested strategy closes the gap between theoretical optimization and real-world application by fusing analytical and deep learning techniques. The findings show that implementing such a system in actual IoT networks is feasible in order to enhance communication efficiency and dependability in crucial healthcare situations, ultimately facilitating prompt emergency responses for long-term patients.

### 3.1 System model and problem formulation

**Fig 1:**
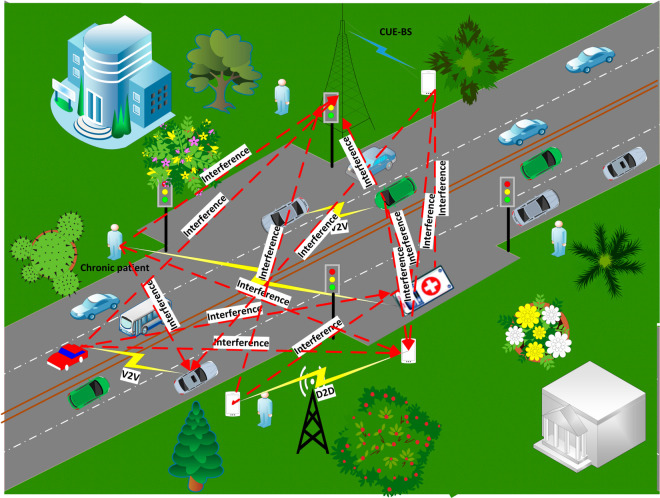
Proposed model.

In the proposed IoT network for chronic patients, it is assumed that a chronic patients (I) with wearable devices (IoT), an emergency vehicle that is always nearby, *K* number of CUEs, base station (BS), *D* number of D2D, which consists of transmitting devices (Dtx) and receiving devices (Drx), and *V* number of V2V, which consists of transmitting vehicles (Vtx) and receiving vehicles (Vrx), share the same spectrum as shown in Figure 1. The communication possibilities include (i) data transmission from the chronic patient’s IoT device to an emergency vehicle; (ii) standard cellular communication in which CUEs speak with BS; (iii) Dtx and Drx engaging in D2D communication; and (iv) Vtx and Vrx engaging in V2V communication. Interference is introduced to emergency vehicles when at least one CUE, transmitting device (Dtx), or transmitting vehicle (Vtx) sharing the same spectrum as the patient’s IoT device has information to send to BS or any receiving devices (Drx). By decreasing interference, the proposed methodology intends to improve communication between patient IoT devices and emergency vehicles. The main goal is to maximize the overall performance of the IoMT network while taking into account the energy efficiency (*EE*) and total achievable data rate (*R*), as expressed by the following equations:

$$\text{Maximize}\;\sum_{i=1}^{I}\sum_{k=1}^{K}\sum_{d=1}^{D}\sum_{v=1}^{V}{EE}_{i,k,d,v}\;\text{Subject to}\;{EE}_{i,k,d,v}:=f_{1}(SINR_{IA},P_{C},P_{D},P_{V})\;\{SINR_{IA}\geq SINR_{th},\;P_{C}\leq P_{Cmax},\;P_{D}\leq P_{Dmax},\;P_{V}\leq P_{Vmax}\}$$ (1)

$$\text{Maximize}\;\sum_{i=1}^{I}\sum_{k=1}^{K}\sum_{d=1}^{D}\sum_{v=1}^{V}R_{i,k,d,v}\;\text{Subject to}\;R_{i,k,d,v}:=f_{2}(SINR_{IA},P_{C},P_{D},P_{V})\;\{SINR_{IA}\geq SINR_{th},\;P_{C}\leq P_{Cmax},\;P_{D}\leq P_{Dmax},\;P_{V}\leq P_{Vmax}\}$$ (2)

where $EE_{i,k,d,v}$ stands for system energy efficiency based on the suggested problem formulation, and $R_{i,k,d,v}$ presents the total achievable data rate. These values are linked to the *k*–*th* path between CUE and BS, the *d*–*th* path between D2D devices, the *v*–*th* path between V2V, and the *i*–*th* path between patient IoT devices and an emergency vehicle. The signal-to-interference-plus-noise ratio for the IoT-to-emergency vehicle link and the necessary system signal-to-interference-plus-noise ratio are symbolized by *SINR*_*IA*_ and *SINR*_*th*_, respectively. Similar to this, *P*_*C*_ and *P*_*Cmax*_ stand for the transmission power of the CUE and its maximum transmission power, whereas *P*_*D*_ and *P*_*Dmax*_ stand for the D2D communication link’s transmission power and its maximum transmission power. Finally, $P_{V}$ and $P_{Vmax}$ represent the vehicle transmission power and maximum vehicle transmission power, respectively.

In order to facilitate the widespread deployment of chronic patients’ IoT, CUE, D2D, and V2V and allow their simultaneous access to the channel, the proposed model [32,33] selects non-orthogonal multiple access (NOMA) as the suitable access mechanism. Additionally, the proposed model operates on the assumption of a Rayleigh fading channel with additive white Gaussian noise (AWGN) [34]. Furthermore, the model assumes that the channel fading coefficients for different transmission connections are statistically independent of one another. Consequently, the following is an expression for the network’s achievable data rate (*R*) and energy efficiency (*EE*):

$$EE=\frac{R_{IA}}{P_{I}+P_{o}}+\frac{R_{CB}}{P_{C}+P_{o}}+\frac{R_{DD}}{P_{D}+P_{o}}+\frac{R_{VV}}{P_{V}+P_{o}}$$ (3)

$$R=R_{IA}+R_{CB}+R_{DD}+R_{VV}$$ (4)

where achievable data rates for the chronic patient IoT devices and the emergency vehicle link, CUE-BS, D2D links, and V2V links which are indicated by the symbols *R*_*IA*_, *R*_*CB*_, *R*_*DD*_, and $R_{VV}$, respectively. *P*_*I*_ and *P*_*o*_ represent the IoT transmission power of chronic patient and the internal circuitry power consumption, respectively. Therefore, *R*_*IA*_, *R*_*CB*_, *R*_*DD*_, and $R_{VV}$ can be expressed by:

$$R_{IA}=B\log_{2}\left(1+\frac{P_{I}H_{IA}}{\sum_{k=1}^{K}P_{C}H_{C_{k}A}+\sum_{d=1}^{D}P_{D}H_{D_{d}A}+\sum_{v=1}^{V}P_{V}H_{V_{v}A}+N}\right)$$ (5)

$$R_{CB}=B\log_{2}\left(1+\frac{P_{C}H_{CB}}{\sum_{i=1}^{I}P_{I}H_{I_{i}B}+\sum_{d=1}^{D}P_{D}H_{D_{d}B}+\sum_{v=1}^{V}P_{V}H_{V_{v}B}+N}\right)$$ (6)

$$R_{DD}=B\log_{2}\left(1+\frac{P_{D}H_{DD}}{\sum_{i=1}^{I}P_{I}H_{I_{i}D}+\sum_{k=1}^{K}P_{C}H_{C_{k}D}+\sum_{v=1}^{V}P_{V}H_{V_{v}D}+N}\right)$$ (7)

$$R_{VV}=B\log_{2}\left(1+\frac{P_{V}H_{VV}}{\sum_{i=1}^{I}P_{I}H_{I_{i}V}+\sum_{k=1}^{K}P_{C}H_{C_{k}V}+\sum_{d=1}^{D}P_{D}H_{D_{d}V}+N}\right)$$ (8)

where *H*_*IA*_, $H_{C_{k}A}$, $H_{D_{d}A}$ and $H_{V_{v}A}$ are the channel gain coefficient between the IoT device of the patient and emergency vehicle, CUE and emergency vehicle, Dtx and emergency vehicle, and Vtx and emergency vehicle, respectively. Symbols *H*_*CB*_, $H_{I_{i}B}$, $H_{D_{d}B}$ and $H_{V_{v}B}$ are the channel gain coefficient between CUE and BS, patient IoT and BS, Dtx and BS, and Vtx and BS, respectively. Symbols *H*_*DD*_, *H*(*I*_*i*_*D*), $H_{C_{k}D}$ and $H_{V_{v}D}$ are the channel gain coefficient between Dtx and Drx, patient IoT and Dtx, CUE and Drx, and Vtx and Drx, respectively. $H_{VV}$, $H_{I_{i}V}$, $H_{C_{k}V}$ and $H_{D_{d}V}$ are the channel gain coefficient between Vtx and Vrx, patient IoT and Vrx, CUE and Vrx, and Dtx and Vrx, respectively. *N* and *B* stand for the noise power and channel system bandwidth, respectively, in this context.

The primary objective of the proposed methodology is to optimize the achievable data rate (R) and total energy efficiency (EE) under various environmental conditions, as stated in equations 1 and 2. As a result, the following formula serves as the Lagrangian for the optimization problem given in Eq 1 and 2:

$$L\{{SINR}_{IA},P_{C},P_{D},P_{V},\lambda_{1},\lambda_{2},\lambda_{3},\lambda_{4}\}=EE+\lambda_{1}({SINR}_{IA}-{SINR}_{th})+\lambda_{2}(P_{C}-P_{Cmax})\;+\lambda_{3}(P_{D}-P_{Dmax})+\lambda_{4}(P_{V}-P_{Vmax}).\;$$ (9)

$$L\{{SINR}_{IA},P_{C},P_{D},P_{V},\mu_{1},\mu_{2},\mu_{3},\mu_{4}\}=R+\mu_{1}({SINR}_{IA}-{SINR}_{th})+\mu_{2}(P_{C}-P_{Cmax})\;+\mu_{3}(P_{D}-P_{Dmax})+\mu_{4}(P_{V}-P_{Vmax}).\;$$ (10)

The Lagrangian multipliers that are not negative in this situation are represented by the symbols $\lambda_{1}$, $\lambda_{2}$, $\lambda_{3}$, $\lambda_{4}$, $\mu_{1}$, $\mu_{2}$, $\mu_{3}$, and $\mu_{4}$. The derivative of Eq 9 with regard to *P*_*I*_, *P*_*C*_, *P*_*D*_, and $P_{V}$ must be taken into consideration in order to determine the values of $\lambda_{1}$, $\lambda_{2}$, $\lambda_{3}$ and $\lambda_{4}$ to fulfil the optimization problem requirements for energy efficiency (EE). Thus, the following can be used to produce $\lambda_{1}$, $\lambda_{2}$, $\lambda_{3}$ and $\lambda_{4}$:

$$\lambda_{1}=(\frac{B}{\left(P_{I}+P_{o}\right)}\cdot X_{1}+\frac{B}{{\left(P_{I}+P_{o}\right)}^{2}\cdot X_{2}}\cdot \log_{2}(1+P_{I}\cdot X_{2})+\frac{B}{(P_{C}+P_{o})\cdot X_{2}}\cdot X_{3}\cdot X_{4}+\;\frac{B}{(P_{D}+P_{o})\cdot X_{2}}\cdot X_{5}\cdot X_{6}+\frac{B}{(P_{V}+P_{o})\cdot X_{2}}\cdot X_{7}\cdot X_{8})$$ (11)

$$\lambda_{2}=\frac{B}{\left(P_{I}+P_{o}\right)}X_{1}\cdot \frac{P_{I}\sum_{k=1}^{K}H_{C_{k}A}\cdot X_{2}}{C}+\frac{B}{{\left(P_{C}+P_{o}\right)}^{2}}\log_{2}(1+P_{C}X_{9})-\frac{B}{\left(P_{C}+P_{o}\right)}X_{3}X_{9}\;+\frac{B}{\left(P_{D}+P_{o}\right)}X_{5}X_{10}+\frac{B}{\left(P_{V}+P_{o}\right)}X_{7}X_{11}+\lambda_{1}\left(\frac{P_{I}\sum_{k=1}^{K}H_{C_{k}A}\cdot X_{2}}{C}\right)$$ (12)

$$\lambda_{3}=\frac{B}{\left(P_{I}+P_{o}\right)}X_{1}\cdot \frac{P_{I}\sum_{l=1}^{L}H_{D_{d}A}\cdot X_{2}}{C}+\frac{B}{\left(P_{C}+P_{o}\right)}X_{3}X_{12}\;\;+\frac{B}{{\left(P_{D}+P_{o}\right)}^{2}}\log_{2}(1+P_{D}X_{13})-\frac{B}{\left(P_{D}+P_{o}\right)}X_{5}X_{13}+\frac{B}{\left(P_{V}+P_{o}\right)}X_{7}X_{14}\;\;+\lambda_{1}\left(\frac{P_{I}\sum_{d=1}^{D}H_{D_{d}A}\cdot X_{2}}{C}\right)$$ (13)

$$\lambda_{4}=\frac{B}{\left(P_{I}+P_{o}\right)}X_{1}\cdot \frac{P_{I}\sum_{v=1}^{V}H_{V_{v}A}\cdot X_{2}}{C}+\frac{B}{\left(P_{C}+P_{o}\right)}X_{3}X_{15}\;\;+\frac{B}{\left(P_{D}+P_{o}\right)}X_{5}X_{16}+\frac{B}{{\left(P_{V}+P_{o}\right)}^{2}}\log_{2}(1+P_{V}X_{17})-\frac{B}{\left(P_{V}+P_{o}\right)}X_{7}X_{17}\;\;+\lambda_{1}\left(\frac{P_{I}\sum_{v=1}^{V}H_{V_{v}A}\cdot X_{2}}{C}\right)$$ (14)

where


$$C=\sum_{k=1}^{K}P_{C}H_{C_{k}A}+\sum_{d=1}^{D}P_{D}H_{D_{d}A}+\sum_{v=1}^{V}P_{V}H_{V_{v}A}+N,$$

$$X_{1}=\frac{\sum_{k=1}^{K}P_{C}H_{C_{k}A}+\sum_{d=1}^{D}P_{D}H_{D_{d}A}+\sum_{v=1}^{V}P_{V}H_{V_{v}A}+N}{\sum_{k=1}^{K}P_{C}H_{C_{k}A}+\sum_{d=1}^{D}P_{D}H_{D_{d}A}+\sum_{v=1}^{V}P_{V}H_{V_{v}A}+N+P_{I}H_{IA}},$$

$$X_{2}=\frac{H_{IA}}{\sum_{k=1}^{K}P_{C}H_{C_{k}A}+\sum_{d=1}^{D}P_{D}H_{D_{d}A}+\sum_{v=1}^{V}P_{V}H_{V_{v}A}+N},$$

$$X_{3}=\frac{\sum_{i=1}^{I}P_{I}H_{I_{i}B}+\sum_{d=1}^{D}P_{D}H_{D_{d}B}+\sum_{v=1}^{V}P_{V}H_{V_{v}B}+N}{\sum_{i=1}^{I}P_{I}H_{I_{i}B}+\sum_{d=1}^{D}P_{D}H_{D_{d}B}+\sum_{v=1}^{V}P_{V}H_{V_{v}B}+N+P_{C}H_{CB}},$$

$$X_{4}=\frac{P_{C}H_{CB}\sum_{i=1}^{I}H_{I_{i}B}}{{\left(\sum_{i=1}^{I}P_{I}H_{I_{i}B}+\sum_{d=1}^{D}P_{D}H_{D_{d}B}+\sum_{v=1}^{V}P_{V}H_{V_{v}B}+N\right)}^{2}},$$

$$X_{5}=\frac{\sum_{i=1}^{I}P_{I}H_{I_{i}D}+\sum_{k=1}^{K}P_{C}H_{C_{k}D}+\sum_{v=1}^{V}P_{V}H_{V_{v}D}+N}{\sum_{i=1}^{I}P_{I}H_{I_{i}D}+\sum_{k=1}^{K}P_{C}H_{C_{k}D}+\sum_{v=1}^{V}P_{V}H_{V_{v}D}+N+P_{D}H_{DD}},$$

$$X_{6}=\frac{P_{D}H_{DD}\sum_{i=1}^{I}H_{I_{i}D}}{{\left(\sum_{i=1}^{I}P_{I}H_{I_{i}D}+\sum_{k=1}^{K}P_{C}H_{C_{k}D}+\sum_{v=1}^{V}P_{V}H_{V_{v}D}+N\right)}^{2}},$$

$$X_{7}=\frac{\sum_{i=1}^{I}P_{I}H_{I_{i}V}+\sum_{k=1}^{K}P_{C}H_{C_{k}V}+\sum_{d=1}^{D}P_{D}H_{D_{d}V}+N}{\sum_{i=1}^{I}P_{I}H_{I_{i}V}+\sum_{k=1}^{K}P_{C}H_{C_{k}V}+\sum_{d=1}^{D}P_{D}H_{D_{d}V}+N+P_{D}H_{DD}},$$

$$X_{8}=\frac{P_{V}H_{VV}\sum_{i=1}^{I}H_{I_{i}V}}{{\left(\sum_{i=1}^{I}P_{I}H_{I_{i}V}+\sum_{k=1}^{K}P_{C}H_{C_{k}V}+\sum_{d=1}^{D}P_{D}H_{D_{d}V}+N\right)}^{2}},$$

$$X_{9}=\frac{H_{CB}}{\sum_{i=1}^{I}P_{I}H_{I_{i}B}+\sum_{d=1}^{D}P_{D}H_{D_{d}B}+\sum_{v=1}^{V}P_{V}H_{V_{v}B}+N},$$

$$X_{10}=\frac{P_{D}H_{DD}\sum_{k=1}^{K}H_{C_{k}D}}{{\left(\sum_{i=1}^{I}P_{I}H_{I_{i}D}+\sum_{k=1}^{K}P_{C}H_{C_{k}D}+\sum_{v=1}^{V}P_{V}H_{V_{v}D}+N\right)}^{2}},$$

$$X_{11}=\frac{P_{V}H_{VV}\sum_{k=1}^{K}H_{C_{k}V}}{{\left(\sum_{i=1}^{I}P_{I}H_{I_{i}V}+\sum_{k=1}^{K}P_{C}H_{C_{k}V}+\sum_{d=1}^{D}P_{D}H_{D_{d}V}+N\right)}^{2}},$$

$$X_{12}=\frac{P_{B}H_{CB}\sum_{l=1}^{L}P_{D}H_{D_{l}B}}{{\left(\sum_{i=1}^{I}P_{I}H_{I_{i}B}+\sum_{d=1}^{D}P_{D}H_{D_{d}B}+\sum_{v=1}^{V}P_{V}H_{V_{v}B}+N\right)}^{2}},$$

$$X_{13}=\frac{H_{DD}}{\sum_{i=1}^{I}P_{I}H_{I_{i}D}+\sum_{k=1}^{K}P_{C}H_{C_{k}D}+\sum_{v=1}^{V}P_{V}H_{V_{v}D}+N},$$

$$X_{14}=\frac{P_{V}H_{VV}\sum_{d=1}^{D}H_{D_{d}V}}{{\left(\sum_{i=1}^{I}P_{I}H_{I_{i}V}+\sum_{k=1}^{K}P_{C}H_{C_{k}V}+\sum_{d=1}^{D}P_{D}H_{D_{d}V}+N\right)}^{2}},$$

$$X_{15}=\frac{P_{B}H_{CB}\sum_{v=1}^{V}P_{V}H_{V_{v}B}}{{\left(\sum_{i=1}^{I}P_{I}H_{I_{i}B}+\sum_{d=1}^{D}P_{D}H_{D_{d}B}+\sum_{v=1}^{V}P_{V}H_{V_{v}B}+N\right)}^{2}},$$

$$X_{16}=\frac{P_{D}H_{DD}\sum_{v=1}^{V}P_{V}H_{V_{v}D}}{{\left(\sum_{i=1}^{I}P_{I}H_{I_{i}D}+\sum_{k=1}^{K}P_{C}H_{C_{k}D}+\sum_{v=1}^{V}P_{V}H_{V_{v}D}+N\right)}^{2}}and$$

$$X_{17}=\frac{H_{VV}}{\sum_{i=1}^{I}P_{I}H_{I_{i}V}+\sum_{k=1}^{K}P_{C}H_{C_{k}V}+\sum_{d=1}^{D}P_{D}H_{D_{d}V}+N}$$

To simplify mathematical statements and prevent duplication, words *X*_1_ through *X*_17_ are utilized as shorthand representations of composite expressions. The variables utilized in these definitions, such as *H*_*IA*_, *P*_*C*_, *P*_*D*_, etc., have previously been presented and discussed earlier in this section. To promote clarity and traceability for the reader, Table 1 (Table of Abbreviations) contains a summary of these variables, as well as their definitions.

**Table 1:** List of variables and abbreviations used in the proposed model.

Symbol	Description
*I*	Number of chronic patients with IoT devices
*K*	Number of cellular user equipment (CUE)
*D*	Number of D2D communication links
*V*	Number of V2V communication links
$D_{\text{tx}},D_{\text{rx}}$	Transmitting and receiving devices in D2D
$V_{\text{tx}},V_{\text{rx}}$	Transmitting and receiving vehicles in V2V
BS	Base Station
*EE*	Energy efficiency
*R*	Total achievable data rate
${\text{SINR}}_{IA}$	Signal-to-Interference-plus-Noise Ratio for IoT–ambulance link
${\text{SINR}}_{th}$	Threshold SINR for reliable communication
*P*_*I*_	Transmission power of IoT device
*P*_*C*_, *P*_*Cmax*_	CUE transmission power and its maximum limit
*P*_*D*_, *P*_*Dmax*_	D2D transmission power and its maximum limit
$P_{V}$, $P_{Vmax}$	V2V transmission power and its maximum limit
*P*_*o*_	Internal circuitry power consumption
*B*	Bandwidth of the communication channel
*N*	Noise power
*H*_*IA*_	Channel gain: IoT to ambulance
$H_{C_{k}A}$	Channel gain: CUE *k* to ambulance
$H_{D_{d}A}$	Channel gain: D2D *d* to ambulance
$H_{V_{v}A}$	Channel gain: V2V *v* to ambulance
*H*_*CB*_	Channel gain: CUE to BS
$H_{I_{i}B}$	Channel gain: IoT *i* to BS
$H_{D_{d}B}$	Channel gain: D2D *d* to BS
$H_{V_{v}B}$	Channel gain: V2V *v* to BS
*H*_*DD*_	Channel gain: D2D transmitter to receiver
$H_{I_{i}D}$	Channel gain: IoT *i* to D2D receiver
$H_{C_{k}D}$	Channel gain: CUE *k* to D2D receiver
$H_{V_{v}D}$	Channel gain: V2V *v* to D2D receiver
$H_{VV}$	Channel gain: V2V transmitter to receiver
$H_{I_{i}V}$	Channel gain: IoT *i* to V2V receiver
$H_{C_{k}V}$	Channel gain: CUE *k* to V2V receiver
$H_{D_{d}V}$	Channel gain: D2D *d* to V2V receiver
*d*_*IA*_	Distance between IoT device and ambulance
*pl*_*o*_	Path loss constant
*α*	Path loss exponent
$\lambda_{1}$ to $\lambda_{4}$	Lagrangian multipliers for EE constraints
$\mu_{1}$ to $\mu_{4}$	Lagrangian multipliers for data rate constraints
*X*_1_ to *X*_17_	Composite auxiliary expressions derived from above variables

Eq 9 can also be derived with respect to *lambda*_1_, *lambda*_2_, *lambda*_3_, and *lambda*_4_. This allows for the determination of the optimal required distance (*d*_*IA*_) between patient IoT devices and the emergency vehicle, as well as the optimal required CUE interfere transmission power (*P*_*C*_), optimal required DTx interfere transmission power (*P*_*D*_), and the optimal required VTx interfere transmission power ($P_{V}$), this can be represented as follows:

$$d_{IA}={\left[\frac{{SINR}_{th}\left(\sum_{k=1}^{K}P_{C}H_{C_{k}A}+\sum_{d=1}^{D}P_{D}H_{D_{d}A}+\sum_{v=1}^{V}P_{V}H_{V_{v}A}+N\right)}{P_{I}/pl_{o}}\right]}^{-1/\alpha }$$ (15)

where *pl*_*o*_ is the constant path loss and *α* is the path loss exponent.

$$P_{C}=P_{C_{max}}$$ (16)

$$P_{D}=P_{D_{max}}$$ (17)

$$P_{V}=P_{V_{max}}$$ (18)

The derivative of Eq 10 with respect to *P*_*I*_, *P*_*C*_, *P*_*D*_, and $P_{V}$ can be used to determine the values of $\mu_{1}$, $\mu_{2}$, $\mu_{3}$, and $\mu_{4}$ in order to meet the constraint of the optimization problem for (*R*). Consequently, $\mu_{1}$, $\mu_{2}$, $\mu_{3}$, and $\mu_{4}$ can be expressed as follows:

$$\mu_{1}=\frac{BX_{1}X_{2}+BX_{3}X_{4}+BX_{5}X_{6}+BX_{7}X_{8}}{X_{2}}$$ (19)

$$\mu_{2}=BX_{1}\left(\frac{P_{I}\sum_{k=1}^{K}H_{C_{k}A}X_{2}}{C}\right)-BX_{3}X_{9}+BX_{5}X_{10}+BX_{7}X_{11}+\lambda_{1}\left(\frac{P_{I}\sum_{k=1}^{K}H_{C_{k}A}X_{2}}{C}\right)$$ (20)

$$\mu_{3}=BX_{1}\left(\frac{P_{I}\sum_{l=1}^{L}H_{D_{d}A}X_{2}}{C}\right)+BX_{3}X_{12}-BX_{5}X_{13}+BX_{7}X_{14}+\lambda_{1}\left(\frac{P_{I}\sum_{d=1}^{D}H_{D_{d}A}X_{2}}{C}\right)$$ (21)

$$\mu_{4}=BX_{1}\left(\frac{P_{I}\sum_{v=1}^{V}H_{V_{v}A}X_{2}}{C}\right)+BX_{3}X_{15}+BX_{5}X_{16}-BX_{7}X_{17}+\lambda_{1}\left(\frac{P_{I}\sum_{v=1}^{V}H_{V_{v}A}X_{2}}{C}\right)$$ (22)

Eq 10 can be derived with respect to *mu*_1_, *mu*_2_, *mu*_3_, and *mu*_4_. This will yield the optimal required interference distance (*d*_*IA*_) between patient IoT devices and emergency vehicles, the optimal required CUE interfere transmission power (*P*_*C*_), the optimal required DTx interfere transmission power (PD), and the optimal required VTx interfere transmission power ($P_{V}$). The overall achievable data rate (*R*), which can be computed as follows, will be optimized as a result.

$$d_{IA}={\left[\frac{{SINR}_{th}\left(\sum_{k=1}^{K}P_{C}H_{C_{k}A}+\sum_{d=1}^{D}P_{D}H_{D_{d}A}+\sum_{v=1}^{V}P_{V}H_{V_{v}A}+N\right)}{P_{I}/pl_{o}}\right]}^{-1/\alpha }$$ (23)

$$P_{C}=P_{C_{max}}$$ (24)

$$P_{D}=P_{D_{max}}$$ (25)

$$P_{V}=P_{V_{max}}$$ (26)

### 3.2 Data generation

The proposed model’s equations (described in System Model and Problem Formulation Section) were implemented using MATLAB simulations, which also provided the necessary dataset. Table 2 displays the values of the simulation parameters. To improve communication between the emergency vehicle and the patient IoT, models that will be deployed on all transmitting devices will be trained with the datasets. The total number of records is 54,848. Each record represents a specific combination of the following: the distances between CUB and BS (*d*_*CB*_), Dtx and Drx (*d*_*DD*_), Vtx and Vrx ($d_{VV}$), the necessary signal-to-interference-plus-noise-ratio threshold (*SINR*_*th*_), the transmission power of the IoT devices (*P*_*I*_), the CUE transmission power (*P*_*C*_), the D2D transmission power (*P*_*D*_), and the V2V transmission power ($P_{V}$).

**Table 2:** Simulation parameters.

Parameter	Value
*N*	-174 dBm/Hz [35]
*B*	10 Mbit/s [36]
*α*	4
*P*_*I*_	23 dBm [37]
*P*_*C*_	23 dBm [37]
*P*_*D*_	23 dBm [37]
$P_{V}$	23 dBm [37]
*SINR*_*th*_	20 dB [37]
Pathloss between CUE and BS	$148+40\log_{2}\left(\frac{d_{CB}}{\text{km}}\right)$
Pathloss between D2D link	$128.1+37.6\log_{2}\left(\frac{d_{DD}}{\text{km}}\right)$
Pathloss between V2V link	$128.1+37.6\log_{2}\left(\frac{d_{VV}}{\text{km}}\right)$

These large datasets were created thanks to the MATLAB simulations, which also made it possible to verify the suggested model’s resilience in various scenarios. The dataset’s varied parameter combinations are meant to replicate real-world situations, where elements like power distribution, ambient interference, and device mobility have a big influence on communication effectiveness. In order to improve system flexibility in dynamic IoT situations, these records were utilized to train machine learning models that can forecast optimal configurations.

**Fig 2:**
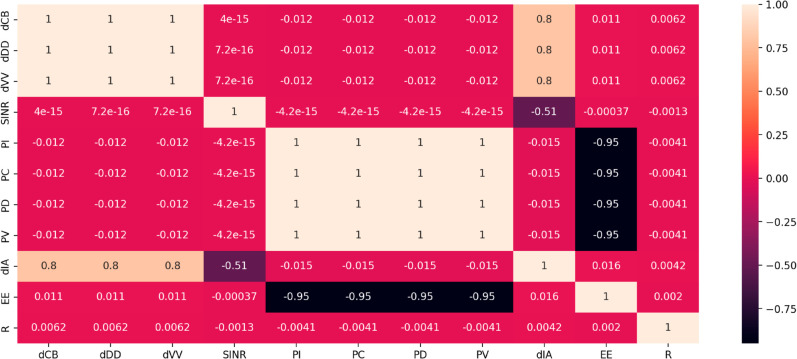
Pearson correlation coefficients of each input parameter (*d*_*CB*_, *d*_*DD*_, $d_{VV}$, *SINR*_*th*_, *P*_*I*_, *P*_*C*_, *P*_*D*_ and $P_{V}$) and the output (*d*_*IA*_, EE and R).

The Pearson coefficients in Figure 2 show the relationship between each input and output parameter. The graph shows that *EE* has a large negative correlation with the *P*_*I*_, *P*_*C*_, *P*_*D*_, and $P_{V}$ parameters, whereas the output *d*_*IA*_ has a high correlation with the *d*_*CB*_, *d*_*DD*_, and $d_{VV}$ parameters. Furthermore, there is little association between parameters *R* and the input parameters. This research shows how crucial distance parameters (*d*_*CB*_, *d*_*DD*_, $d_{VV}$) are to sustaining strong communication links and emphasizes the significance of optimizing power parameters (*P*_*I*_, *P*_*C*_, *P*_*D*_, and $P_{V}$) to increase energy efficiency (*EE*). Knowing these relationships aids in improving the model to guarantee dependable data transfer while reducing energy usage, which is crucial for Internet of Things-based emergency response systems.

### 3.3 Proposed deep learning model

**Fig 3:**
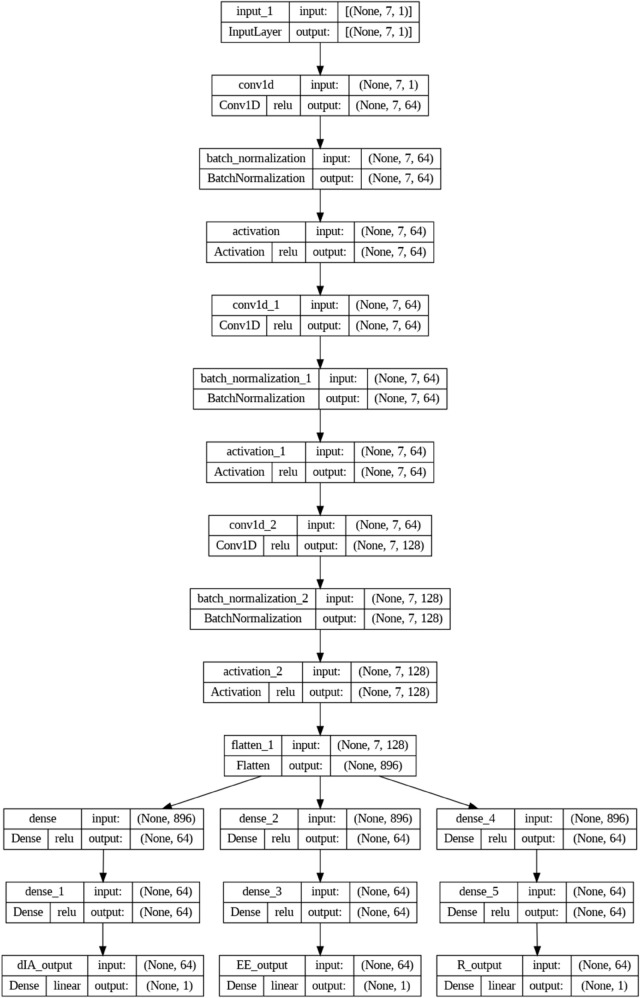
Proposed deep learning model.

The proposed deep learning model is introduced and explained in this section. To aid in the learning of the model weights, a normalization step must be finished prior to incorporating the variables into the suggested deep learning model. Prior to being incorporated into the model, each variable is normalized using the min-max scaling technique. The output parameters, *d*_*IA*_, EE, and R, are generated from the last dense layer using the eight input variables, *d*_*CB*_, *d*_*DD*_, $d_{VV}$, *SINR*_*th*_, *P*_*I*_, *P*_*C*_, *P*_*D*_, and $P_{V}$. As seen in Figure 3, the model is composed of three separate phases: thick layers, flattening, and 1D-CNN. Three 1D-CNN layers, each with 64, 64, and 128 filters in addition to a size 1 kernel, process the normalized input parameters.

A grid search was performed to test several choices before determining the number of nodes for the dense layers and the number of filters for the 1D-CNN. The Rectified Linear Unit (ReLU) has been used as the activation function for all hidden layers, and the grid search took activation function selection into account. The flattening layer is followed by six dense layers, which produce the regression result. A grid search was used to test a number of options prior to deciding on the number of nodes for the dense layers and the number of filters for the 1D-CNN. The Rectified Linear Unit (ReLU) has been used as the activation function for all hidden layers. The grid search also considered the choice of activation functions and experimented with several techniques that may be used to monitor the hidden layers in the suggested model. Each hidden layer’s output was fed into a parametric rectified linear unit (PReLU) activation function to produce the best results.

In order to prevent any one variable from unduly affecting the model’s learning process, this method highlights the significance of preprocessing through normalization to guarantee that all input features are scaled to a consistent range. The 1D-CNN layers are made to successfully learn spatial hierarchies in order to identify complex patterns and relationships in the normalized input. The model may abstract high-level information and improve predictions through regression thanks to the usage of many thick layers after the CNN stages. Grid search is used for hyperparameter tuning, which optimizes the architecture to strike a compromise between performance accuracy and computing economy. By avoiding problems like vanishing gradients, the model’s robust learning is ensured by the experimentation with activation functions, such as ReLU and PReLU. CNN layers and dense layers work together to produce a versatile yet strong architecture that can accurately anticipate the output parameters by processing intricate correlations between the eight input variables. In order to manage the subtleties of IoT communication systems, this deep learning framework was meticulously designed. This ensures that the outputs are not only correct but also robust to changes in the input parameters. The model’s performance and stability are further enhanced by the application of ReLU and PReLU, which guarantee that the model can generalize to previously unknown data.

With the mean absolute error (MAE) loss function and root mean squared error (RMSE) as goals, the adaptive moment (Adam) is the optimization employed in the suggested model. Depending on the learning process, Adam can learn the necessary parameters in an adaptable manner. MAE calculates the average difference between actual and expected values, whereas RMSE is the root square of the average of the squared discrepancies between real and anticipated values. These can be expressed as:

$$MAE=\frac{\sum_{j=1}^{n}|y_{j}-x_{j}|}{n}$$ (27)

$$RMSE=\sqrt{\frac{\sum_{j=1}^{n}(y_{j}-x_{j}{)}^{2}}{n}}$$ (28)

where *n* is the total amount of data gathered, *y*_*j*_ is the actual value, and *x*_*j*_ is the forecast value. The following part details the experiments that were carried out in order to test, assess, and train the suggested model.

## 4 Results

This section displays the performance of the proposed deep learning and analytical models. Additionally, MATLAB and Python simulations were used to evaluate the success of the proposed approach in terms of optimized energy efficiency and achievable data rate. In order to verify the usefulness of the suggested models in practical situations, the performance evaluation primarily compares their outputs with theoretical benchmarks. A thorough examination of the analytical model’s ability to determine ideal parameters was made possible by the use of MATLAB simulations to model different IoT network setups and interference situations. These simulations offered insightful information about how various setups affect data rates and energy consumption in various scenarios. Combining simulations in Python and MATLAB A thorough evaluation framework is provided by the combination of MATLAB and Python simulations. Python-based simulations show the deep learning model’s versatility and practical application in dynamic contexts, while MATLAB simulations offer a thorough understanding of the underlying theoretical model. This two-pronged strategy guarantees that the suggested method can function reliably in actual IoT networks in addition to being conceptually sound.

**Fig 4:**
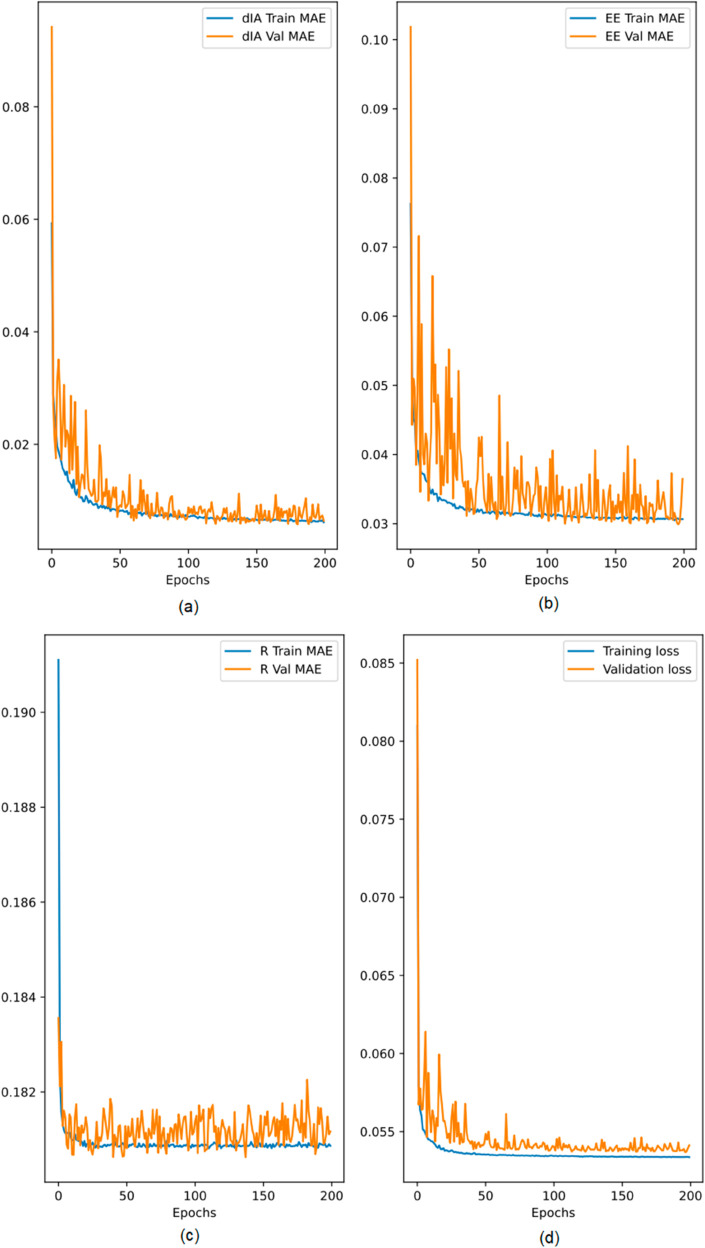
Training and validation mean absolute error generated during training the proposed model.

The proposed deep learning model outlined in Proposed Deep Learning Model section is tested and evaluated, as shown in Figure 4. The datasets were split into an 80% train set and a 20% test set. Figures 4(a), 4(b), and 4(c) show the training and validation mean absolute errors for the required *d*_*IA*_, EE, and R, respectively. All of these graphs show that the results were little changing after epoch 100, indicating that additional training was not required. Additionally, the independent training and validation errors for each output were about similar, as shown in Figure 4(d), suggesting that the proposed model was neither overfitted nor underfitted. Additionally, it demonstrates how the loss of separate training and validation mistakes diminishes and stabilizes at a specific point. The model architecture’s resilience and capacity to extract significant patterns from the data are demonstrated by the consistency between the two sets of errors.

Furthermore, the steady decline in mistakes shown in the early epochs illustrates how well the model learn, with the optimization algorithms successfully modifying the weights to reduce inconsistencies. In order to prevent problems like excessive training or stagnation at unsatisfactory performance, the model’s convergence to an optimal solution is further confirmed by the stabilization of loss values at a certain point. The findings also highlight how important it was to choose the right hyperparameters, including the number of epochs, in order to achieve the stability that was shown without wasting any processing power. This assessment offers compelling proof of the model’s capacity to achieve the required accuracy while preserving computational effectiveness, which qualifies it for real-time Internet of Things applications. These results show that the suggested deep learning model is successful in producing precise and consistent predictions for the crucial outputs *d*_*IA*_, EE, and R. Figure 4 illustrates the model’s performance, which demonstrates its capacity to handle the intricate needs of IoT-based communication systems while guaranteeing dependable results for real-world implementations.

For the analytical and deep learning models, all simulation parameters were chosen to represent genuine scenarios in emergency healthcare communication settings. The transmission power for IoT devices (*P*_*I*_), cellular users (*P*_*C*_), D2D devices (*P*_*D*_), and vehicles ($P_{V}$) was universally set to 23 dBm, as per the configuration used in [37], which reflects the highest permitted transmission power in many LTE/5G systems and medical-grade IoT devices. The SINR threshold was adjusted to 20 dB to align with the performance requirements necessary for ultra-reliable low-latency communication (URLLC) in critical circumstances such as emergency patient monitoring [37]. To simulate realistic wireless propagation, we adopted standard path loss models:

- CUE to BS: $PL_{CB}(d)=148+40\log_{2}\left(\frac{d_{CB}}{\text{km}}\right)$
- D2D link: $PL_{DD}(d)=128.1+37.6\log_{2}\left(\frac{d_{DD}}{\text{km}}\right)$
- V2V link: $PL_{VV}(d)=128.1+37.6\log_{2}\left(\frac{d_{VV}}{\text{km}}\right)$

These models are based on widely established 3GPP channel models and are appropriate for simulating route loss in both urban vehicle contexts and dense IoT healthcare settings. Their participation validates our simulation methodology for deploying energy-aware healthcare communication systems.

**Fig 5:**
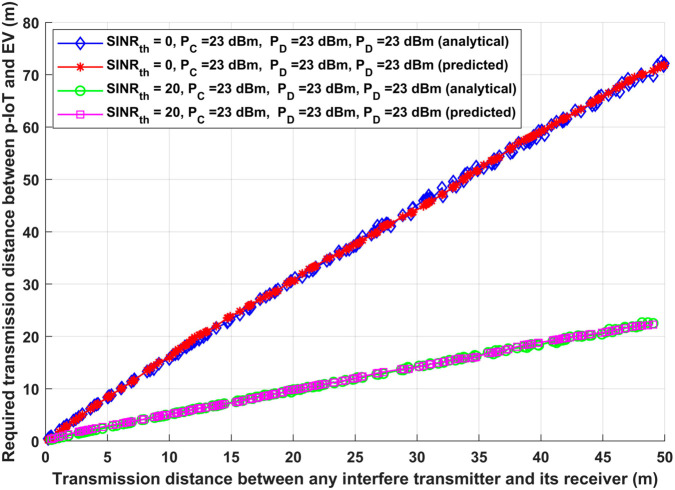
Transmission distance between any interfere transmitter and its receiver and required transmission distance between p-IoT and EV (*d*_*IA*_).

Figure 5 shows the transmission distance between any interfere transmitter and its receiver (D2D links, V2V links, and CUE-BS link) versus the required transmission distance (*d*_*IA*_) between patient IoT devices (p-IoT) and emergency vehicles (EV) using the same patient IoT transmission power (*P*_*I*_), CUE transmission power (*P*_*C*_), D2D transmission power (PD), and V2V transmission power ($P_{V}$). Two different *SINR*_*th*_ of 0 dB and 20 dB have been considered in order to evaluate the efficacy of the proposed model. Furthermore, it has been assumed that all of the interfere devices sent data with the maximum interfere transmission power of 23 dBm, and that the CUE-BS transmission distance (*d*_*CB*_) is five times longer than the transmission distances of D2D and V2V links (*d*_*DD*_ and $d_{VV}$). In the worst case scenario, high amounts of interference transmission power could affect patient IoT transmission data. For any interfere transmission distance and for each *SINR*_*th*_ given, as shown in Figure 5, there is an ideal necessary transmission distance between p-IoT and EV (*d*_*IA*_) to match the needed IoT system performance for both the analytical and deep learning models. To fulfil IoT system performance, for example, the optimal required transmission distance between p-IoT and EV (*d*_*IA*_) for the analytical and deep learning models, respectively, is 69.8121 m and 70.77967 m when *SINR*_*th*_ is 0. On the other hand, the ideal required transmission distance between p-IoT and EV (*d*_*IA*_) is 22.4985 m for the analytical model and 22.25261 m for the deep learning model when *SINR*_*th*_ is 20. These findings are achieved when the interference transmission distance between the CUE-BS connection and the D2D and V2V link is 245.3 m and 49.06 m, respectively.

It is clear from comparing the two cases that a considerable reduction in the needed gearbox distance (*d*_*IA*_) between p-IoT and EV occurs when *SINR*_*th*_ is increased. With a high degree of reliability, this reduction guarantees that the data is sent over a more effective communication channel. Reliable data reception is essential for facilitating efficient decision-making in real-time situations, particularly in applications related to emergency healthcare. These results show how well the analytical and deep learning models adjust to different *SINR*_*th*_ and interference levels. They emphasize how crucial it is to optimize transmission lengths in order to improve the efficiency of IoT systems, especially in high-stakes applications like emergency medical care. The suggested models successfully handle the difficulties brought on by interference and different system needs through this comparative analysis, offering a framework for establishing dependable and effective IoT communication.

**Fig 6:**
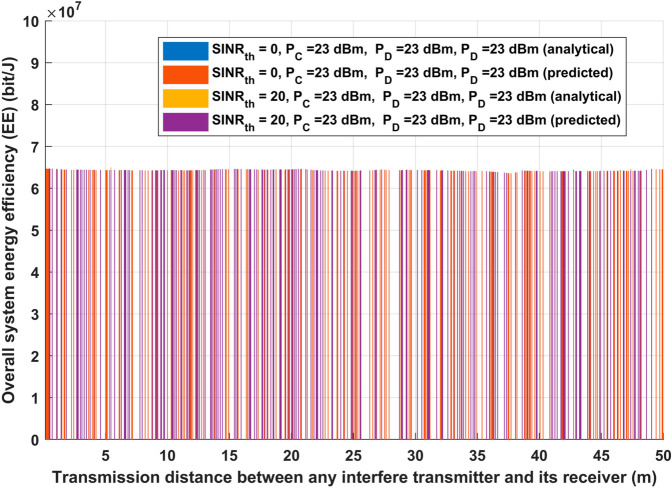
Transmission distance between any interfere transmitter and its receiver vs Overall system energy efficiency (EE).

**Fig 7:**
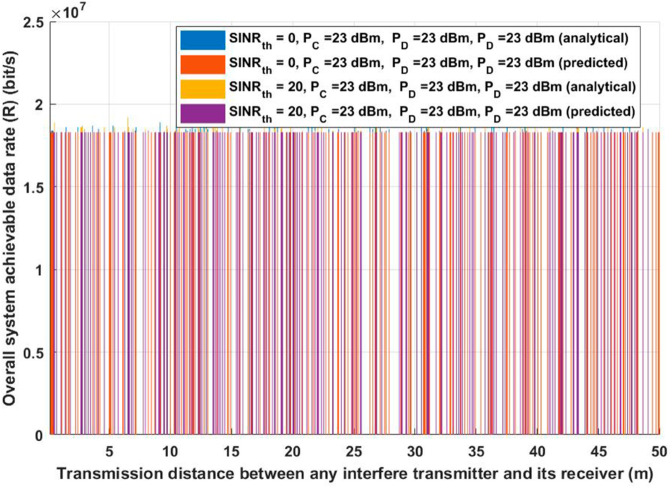
Transmission distance between any interfere transmitter and its receiver vs Overall system achievable data rate (R).

Additionally, for the same two considered scenarios, the suggested approach was evaluated in terms of total system EE and overall achievable data rate, as shown in Figures 6 and 7. As shown in Figure 6, extending the interfere transmission distance while maintaining the same transmission power while changing *SINR*_*th*_ must result in a whole received signal, proving that the conclusions from Figure 6 will help keep the system EE unchanged for both analytical and deep learning model. However, For both the analytical and deep learning methods, the evaluated EE will remain constant as the interference transmission distance increases, thanks to the suggested model, which improves IoT communication by avoiding or reducing interference. Interestingly, even when the interference transmission distance rises, the estimated EE stays constant. The capacity of the suggested model to improve IoT connectivity by preventing or greatly reducing interference from nearby transmitters is responsible for this stability. The suggested method guarantees consistent energy efficiency by dynamically optimizing the system’s parameters. This is crucial for applications involving emergency healthcare IoT devices that require high reliability and low energy usage.

Figure 7 shows similar tendencies when examining the system’s performance in terms of the achievable data rate (R). The suggested model satisfies or exceeds the required system performance requirements under various environmental situations, as the figure attests. The resilience of the concept is demonstrated by the fact that the system’s data rate is unaffected by changes in the interference transmission distance. This result highlights the resilience of the suggested strategy, which successfully guarantees continuous data transfer between IoT devices and emergency vehicles regardless of outside interference sources. Furthermore, Figure 7 highlights how the suggested model can adjust to different interference levels and environmental circumstances without affecting system performance. The model guarantees that the intended levels of data transmission and reception are attained by optimizing IoT communication. In situations requiring high-stakes applications, like emergency healthcare, where constant and dependable communication might directly affect patient outcomes, this skill is essential. In conclusion, the suggested method guarantees a dependable data rate independent of interference transmission distance in addition to achieving consistent energy efficiency. These outcomes demonstrate how well the model handles the twin goals of maximizing energy use and preserving reliable communication in Internet of Things networks. The suggested paradigm lays the groundwork for scalable, effective, and dependable IoT systems in crucial healthcare settings by accomplishing these objectives.

Consideration should be given to the strategic placement of other potentially interfering devices in scenarios involving diverse IoT devices responsible for data transmission, based on the observations derived from Figures 6 and 7, as inferred from the insights provided by Figure 5. This becomes essential in the endeavor to stop, get rid of, or efficiently handle interference. In an emergency, when the patient’s safety and well-being are of utmost importance, this tactical consideration is essential. In addition, it helps ensure data accuracy, which makes well-informed decisions easier to make. The total reliability of the data collected can be improved by carefully managing the location of interfering devices, which lays the groundwork for crucial decision-making in emergency situations.

**Fig 8:**
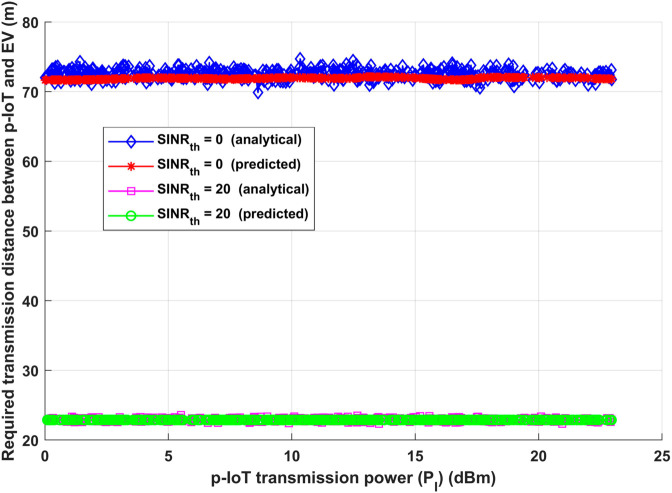
p-IoT transmission power (*P*_*I*_) vs required transmission distance between p-IoT and EV (*d*_*IA*_).

The system is evaluated by determining the optimum transmission distance needed between the patient IoT (p-IoT) and the Emergency Vehicle (EV) under two different Signal-to-Interference-plus-Noise Ratio (*SINR*_*th*_) thresholds (0 dB and 20 dB), considering different p-IoT transmission power levels. We make the assumption that the interference transmission distances for CUE-BS are 250 m in this assessment, and 50 m for D2D and V2V links. Notably, all of the interfering devices’ interference transmission powers constantly align with the p-IoT transmission power. Examining Figure 8, one can see a trend that stands out: a rise in p-IoT transmission power, which indicates that all interfering devices under both *SINR*_*th*_ situations are experiencing a simultaneous increase in transmission power. Remarkably, p-IoT and EV require a consistent ideal transmission distance to sustain the necessary system performance for both analytical and deep learning model. This remark emphasizes how resilient the system is to changes in transmission power and how well it can continuously achieve performance standards. Moreover, it is crucial to emphasize that a decrease in *SINR*_*th*_ corresponds to a corresponding increase in the ideal transmission distance needed between p-IoT and EV. For information to be transmitted accurately and efficiently, this phenomena is essential. To put it simply, when *SINR*_*th*_ drops, the system makes up for it by increasing the transmission distance, which keeps information exchange effective and enhances the system’s overall dependability and efficiency under a variety of circumstances.

**Fig 9:**
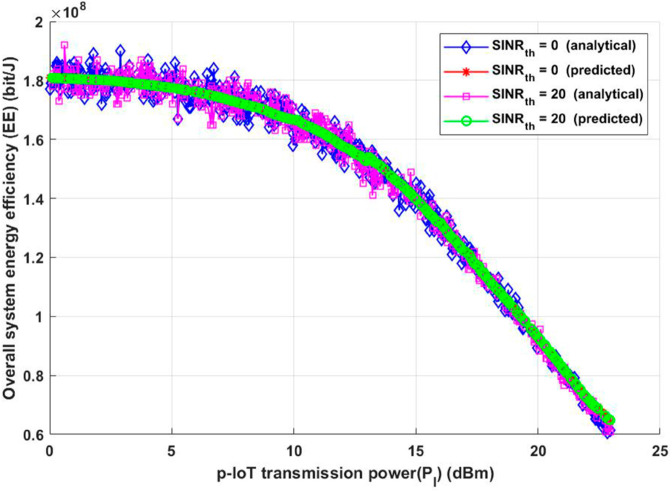
p-IoT transmission power (*P*_*I*_) vs Overall system energy efficiency (EE).

The relationship between patient IoT (p-IoT) transmission power and total system energy efficiency is shown in Figure 9 for two different value for *SINR*_*th*_ as previously mentioned. In this case, a carefully assumed equivalence between p-IoT and interference transmission powers accounts for the illustrated drop in energy efficiency as p-IoT transmission power increases for both analytical and deep learning model. According to this supposition, every increase in p-IoT transmission power is directly reflected in the power of interference transmission for both analytical and deep learning model, leading to a situation where both powers increase simultaneously. A crucial trade-off is introduced by this correlation in the dynamics of the system. Increasing the power of p-IoT transmission can be beneficial in improving communication reliability and signal strength, on the one hand. However, this rise also directly leads to increased interference, which could jeopardise the system’s overall effectiveness. It becomes clear that maintaining this trade-off requires a delicate balance, highlighting the necessity of making strategic decisions while deciding on the best transmission power levels. Practically speaking, this occurrence emphasizes how crucial it is to take into account both the advantages of higher p-IoT transmission power as well as the difficulties brought on by enhanced interference. It is up to system designers and operators to manage this trade-off and find an equilibrium that maximizes energy savings without compromising information transfer accuracy and dependability. Because of this, the observed decline in energy efficiency is a subtle signal that should be investigated further to learn more about the intricate details of interference control and power management in the system architecture.

**Fig 10:**
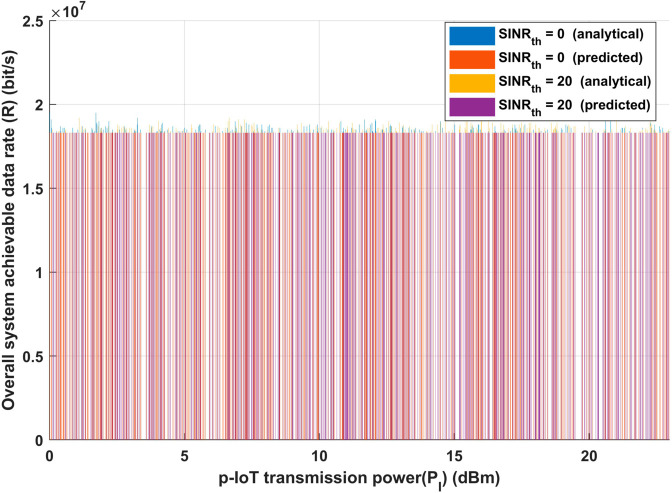
p-IoT transmission power (*P*_*I*_) vs Overall system achievable data rate (R).

Figure 10 illustrates the relationship between the total achievable data rate and p-IoT transmission power for the same hypothetical scenario as Figure 9 describes. Figure 10 shows that even with an increase in P-IoT transmission power, the total achievable data rate is fixed for both analytical and deep learning model. A dynamic balance exists in the system when the constant achievable data rate is maintained throughout a range of patient IoT (p-IoT) transmission powers. Signal strength improves with increased p-IoT transmission power, which could lead to higher data speeds. However, because p-IoT and interference transmission strengths are assumed to be equal, this beneficial benefit is offset by higher interference. The system’s capacity to keep data rates consistent points to an effective adaptation mechanism. This probably incorporates modulation systems, interference mitigation strategies, or dynamic resource allocation. The discovery emphasizes how crucial it is to optimize system parameters in order to balance the advantages of increased signal strength with the difficulties posed by interference. In conclusion, the consistent achievable data rate indicates a robust system that can adjust to variations in p-IoT transmission power through careful management of interference and signal augmentation problems.

**Fig 11:**
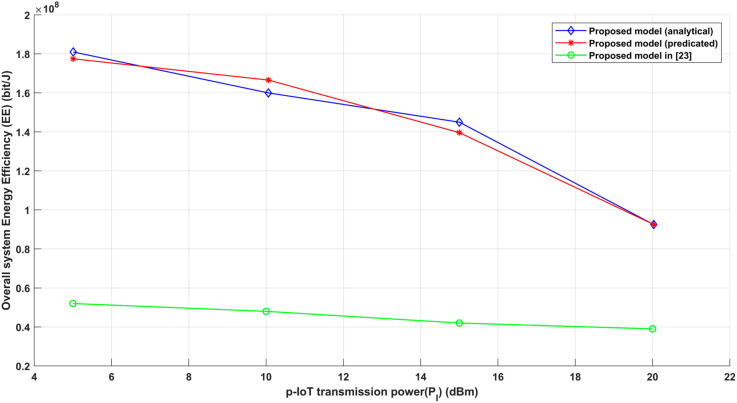
p-IoT transmission power (*P*_*I*_) vs Overall energy efficiency (EE).

In the context of Internet of Things (IoT) communication systems for healthcare applications, the proposed model’s effectiveness and improved performance have been demonstrated by comparing it with an existing technique [23]. Figure 11 provides a thorough comparison of the suggested model with the one described in [23], which investigates the connection between gearbox power and overall energy efficiency. In terms of overall energy efficiency, this comparison demonstrates the obvious benefits of the suggested strategy. The findings show that the recommended approach performs better than the alternative in important energy-related parameters, making it a better option for IoT networks functioning in vital healthcare settings. A number of important aspects contributed to the suggested method’s enhanced performance. First, the proposed method probably makes use of more sophisticated algorithms or methods to figure out the best gearbox distance between emergency vehicles (EV) and patient IoT devices (p-IoT). This optimization procedure is intended to optimize attainable data throughput and energy efficiency, resulting in a more effective use of the resources at hand. The model ensures reliable communication quality while minimizing needless energy use by accurately determining the optimal transmission distance. To assess the efficacy of the suggested model, we compared it to benchmark techniques described in [38]. The performance comparison is described in Table 3, which highlights the gains made by our strategy in important measures.

**Table 3:** Comparison of the proposed model with existing benchmark methods.

EE bit/Joule				
$P_{max}$	Model			
	NOMA-EE-EQ NOMA-EE	DC	FTPA OFDMA	Proposed Model
0.08	4.4*10^7^	3.74*10^7^	1.49*10^7^	1.0142*10^8^
0.123	3.25*10^7^	2*10^7^	1.25*10^7^	7.9786*10^7^
0.1714	2.5*10^7^	1.515*10^7^	1.15*10^7^	6.6217*10^7^
0.2188	2.1*10^7^	1.4*10^7^	1*10^7^	5.6607*10^7^
0.25	1.575*10^7^	1.25*10^7^	9*10^6^	5.2895*10^7^
0.2884	1.51*10^7^	1.11*10^7^	7.5*10^6^	4.6321*10^7^
0.3388	1.4*10^7^	9*10^6^	6*10^6^	4.1935*10^7^
0.3715	1.25*10^7^	7.5*10^6^	5*10^6^	3.8435*10^7^
0.4266	1.15*10^7^	6.5*10^6^	4*10^6^	3.4718*10^7^
0.4742	1*10^7^	5*10^6^	3*10^6^	3.1231*10^7^
0.5	8.8*10^6^	4*10^6^	2*10^6^	3.0773*10^7^

Table 3 compares the energy efficiency (EE) of the proposed model to five benchmark methods: NOMA-EE, NOMA-EE-EQ, DC, FTPA, and OFDMA, across a range of maximum transmission power values (*P*_*max*_). The EE, measured in bits per joule, illustrates how effectively each model transmits data in relation to the energy it consumes—a critical parameter in power-constrained systems such as IoT, automotive networks, and smart healthcare.

- NOMA-EE (Non-Orthogonal numerous Access for Energy Efficiency): This technology use power-domain multiplexing to allow numerous users to share the same frequency. It dynamically modifies power distribution to maximize EE, hence increasing spectral efficiency. However, its performance may suffer under strong inter-user interference or rapidly changing channels.
- NOMA-EE-EQ (NOMA with Equivalent Power Allocation for Energy Efficiency): This simplified version of NOMA-EE assigns equal transmit power to all users regardless of channel circumstances. While it minimizes processing complexity and prevents user prioritizing, it frequently leads in inefficient energy use, particularly in diverse network settings.
- Dynamic Clustering (DC) creates adaptive groups of users or nodes depending on real-time parameters like signal strength and proximity. Power and resource allocation are then optimized within each cluster. Although clustering is useful for reducing interference in dense installations, it adds coordination effort and may limit scalability.
- FTPA (Fractional Transmit Power Allocation) distributes transmit power based on a user’s channel gain. It promotes justice and energy efficiency by providing weaker users somewhat more power. However, it does not account for interference or network dynamics, limiting its usefulness in crowded or high-mobility circumstances.
- OFDMA (Orthogonal Frequency Division Multiple Access) allocates orthogonal subcarriers to each user, resulting in interference-free transmission. It is widely deployed in LTE and 5G networks. Despite its dependability, it lacks the flexibility and spectrum reuse efficiency of NOMA and can be wasteful in terms of energy usage, especially in sparse or uneven traffic situations.

Table 3 shows that the proposed model continuously outperforms benchmark approaches at all *P*_*max*_ levels. At *P*_*max*_ = 0.08, the suggested model’s energy efficiency (EE) achieves 1.0142×10^8^ bit/Joule, which is more than 2.3× greater than NOMA-EE (4.4×10^7^), 2.7× higher than OFDMA (3.74×10^7^), and 6.8× higher than FTPA. NOMA-EE-EQ, which is based on equal power allocation, similarly results in much reduced EE at this level. At a mid-range power level (*P*_*max*_=0.25), the suggested model achieves an EE of 5.2895×10^7^ bit/Joule, surpassing NOMA-EE (1.575×10^7^) by more than 3.3×, OFDMA (1.25×10^7^) by over 4.2×, and FTPA (9×10^6^) by over 5.9×. Even at higher transmission power levels, when EE normally decreases, the suggested model retains a significant advantage. For example, at *P*_*max*_ =0.5, it records an EE of 3.0773×10^7^ bit/Joule, whereas NOMA-EE decreases to 8.8×10^6^, OFDMA to 4×10^6^, and FTPA to just 2×10^6^, making the suggested approach 3.5×, 7.7×, and 15.4× more efficient. As transmission power grows, EE decreases for all models—a common occurrence owing to power saturation and decreased data rate improvements. However, the suggested model shows a slower rate of decrease, resulting in higher EE values overall. This resilience stems from its adaptive and learning-based power regulation method. These findings support the suggested model’s ability to achieve high levels of energy efficiency. Its capacity to manage power adaptively, improve transmission conditions, and outperform both classic and advanced approaches makes it an excellent option for deployment in energy-constrained, high-demand communication environments.

To ensure the suggested 1D-CNN model’s robustness and consistency, we performed a thorough evaluation over five separate runs, each with a distinct random seed. This method allows us to account for potential variability in training caused by stochastic factors like weight initialization and data shuffling. For each run, we measured the Mean Absolute Error (MAE) and Mean Squared Error (MSE) of the three output variables (*d*_*IA*_, EE, and R). We then calculated the mean, standard deviation, and 95% confidence intervals (CI) for each statistic across all runs. These statistical measurements help to understand the model’s dependability, stability, and generalizability. Table 4 summarizes these findings, illustrating that the model consistently performs well with little variance across multiple executions. These findings indicate the model delivers consistent and trustworthy predictions throughout different training sessions, with narrow confidence intervals and little volatility in performance.

**Table 4:** Statistical evaluation of the proposed 1D-CNN model.

Metric	Output	Mean	Std	95% CI
MAE	*d*_*IA*_	0.01443	0.00600	(0.00698, 0.02188)
MAE	EE	0.0177 5	0.00863	(0.00704, 0.02846)
MAE	R	0.01425	0.00690	(0.00568, 0.02282)
MSE	*d*_*IA*_	0.00036	0.00024	(0.00007, 0.00065)
MSE	EE	0.00052	0.00036	(0.00007, 0.00097)
MSE	R	0.00036	0.00031	(−0.00002, 0.00075)

**Fig 12:**
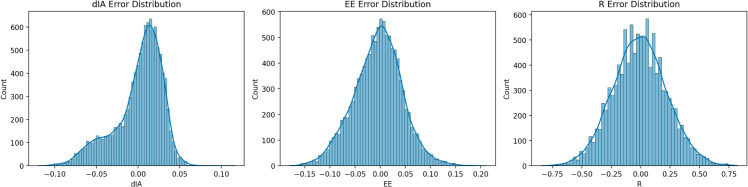
Error distribution histograms for each output parameter: (a) *d*_*IA*_, (b) EE, and (c) R.

In addition to typical assessment measures like MAE and MSE, we looked at the distribution of prediction errors for each of the three output variables *d*_*IA*_, EE, and R to determine our deep learning model’s dependability and consistency. Figure 12 depicts residual histograms (i.e., the difference between real and projected values) for each output, which are overlaid with kernel density estimation (KDE) curves to indicate the distribution shape. The error distribution for *d*_*IA*_ (Figure 12a) is closely centered around zero and is roughly symmetric, indicating that the model does not greatly overestimate or underestimate the output. Similarly, the EE error distribution (Figure 12b) is somewhat right-skewed but remains centered near zero, indicating a minor bias with a few larger error values. R (Figure 12c) has a larger distribution, with a longer tail on both sides, but it is generally balanced around zero. This implies that, while the model’s forecast for R is more variable, it does not consistently diverge in one way. These findings supplement the previously reported MAE, MSE, and confidence interval results by providing visual and statistical evidence that the model errors are stable and well-behaved. Importantly, this confirms that the model generalizes efficiently across test samples and is unlikely to provide unpredictable or distorted predictions.Including error distributions provides a measure of variability and uncertainty around the model’s outputs, which directly addresses concerns about prediction robustness in deep learning models for real-time healthcare systems.

A comprehensive statistical analysis was conducted in Table 5 of the model outputs *d*_*IA*_, EE, and R by varying critical wireless parameters such as Signal-to-Interference-plus-Noise Ratio (SINR), maximum transmission power (*P*_*max*_), and communication distances. We calculated the mean, standard deviation, and 95% confidence intervals for each condition to assess the variability and uncertainty of the data. The results show that when SINR increases, the required transmission distance falls dramatically, indicating improved connection dependability and quicker data delivery. For example, for SINR = 20 dB and *d*_*CB*_ = 50 m, *d*_*DD*_ = 10 m, and $d_{VV}$ = 10 m, the average optimal transmission distance is 5.05 m, as opposed to 15.97 m when SINR = 0 dB. Similarly, altering the communication distance revealed that longer distances result in higher delay and lower reliability, with the required transmission distance increasing from 9.48 m at 50 m to 43.06 m at 250 m under fixed power and SINR. Furthermore, energy efficiency (EE) remained essentially steady throughout situations, with slight gains at shorter distances and higher SINR values, indicating better channel conditions. Importantly, the inclusion of confidence intervals and standard deviations demonstrates that the model predictions are not only accurate but also consistent across numerous situations. This extensive variability study tackles uncertainty problems that are commonly missed in comparable research, improving the model’s interpretability and dependability. The findings demonstrate the system’s flexibility and robustness in a variety of communication contexts common in emergency healthcare and autonomous IoT applications.

**Table 5:** Statistical summary (mean, std, 95% CI) of *dIA*, *EE*, and *R* under varying channel conditions.

Scenario	Metric	Mean	Std	95% CI (Lower)	95% CI (Upper)
**Varying Distance**					
(SINR=20 dB, *P*_*C*_=23 dBm)	dIA	11.8351	6.5436	11.3906	12.2795
	EE	61118922.1557	1211238.8519	61036647.6334	61201196.6780
	R	18295209.5808	391518.0416	18268615.3548	18321803.8069
(SINR=0 dB, *P*_*C*_=23 dBm)	dIA	37.4213	20.6913	36.0158	38.8268
	EE	61162275.4491	1256876.3747	61076900.9557	61247649.9425
	R	18317724.5509	364487.2168	18292966.4187	18342482.6831
**Varying SINR**					
(*d*_*CB*_=50 m, *d*_*DD*_=10 m, $d_{VV}$=10 m, *P*_*C*_=23 dBm)	dIA	9.4816	3.1186	9.3449	9.6184
	EE	61090154.9225	1235094.3389	61036006.2790	61144303.5661
	R	18305347.3263	374588.2964	18288924.7362	18321769.9165
(*d*_*CB*_=250 m, *d*_*DD*_=50 m, $d_{VV}$=50 m, *P*_*C*_=23 dBm)	dIA	43.0563	14.1781	42.4347	43.6779
	EE	61116791.6042	1267977.8788	61061201.2901	61172381.9183
	R	18311794.1029	387647.8164	18294798.9611	18328789.2448
**Varying Power**					
(*d*_*CB*_=50 m, *d*_*DD*_=10 m, $d_{VV}$=10 m, SINR=0 dB)	dIA	15.9669	0.1508	15.9607	15.9731
	EE	145531334.2025	36438727.6707	144041693.2966	147020975.1084
	R	18307518.4702	376205.2692	18292138.9333	18322898.0072
(*d*_*CB*_=50 m, *d*_*DD*_=10 m, $d_{VV}$=10 m, SINR=20 dB)	dIA	5.0480	0.0451	5.0462	5.0499
	EE	145426727.5098	36445159.7923	143936823.6542	146916631.3654
	R	18313211.6471	366557.6448	18298226.5119	18328196.7823
**Varying Power**					
(*d*_*CB*_=250 m, *d*_*DD*_=50 m, $d_{VV}$=50 m, SINR=0 dB)	dIA	72.4889	0.6649	72.4617	72.5160
	EE	145506779.6610	36422879.5404	144017786.6379	146995772.6842
	R	18301999.1308	368764.5673	18286923.7750	18317074.4866
(*d*_*CB*_=250 m, *d*_*DD*_=50 m, $d_{VV}$=50 m, SINR=20 dB)	dIA	22.9224	0.2118	22.9137	22.9310
	EE	145400738.8092	36374814.3204	143913710.7262	146887766.8923
	R	18312516.2973	371213.9490	18297340.8090	18327691.7855

Additionally, the suggested approach’s intrinsic flexibility enables a dynamic adjustment of gearbox power and distance, guaranteeing that energy efficiency is maintained even in the face of shifting environmental conditions. This flexibility plays a major role in the model’s capacity to optimize both transmission distance and signal quality in order to increase energy efficiency. In addition to the optimization methodologies, the fundamental architectural or design ideas underlying the proposed methodology may naturally contribute to enhanced energy efficiency. Novel methods to important elements including modulation schemes, transmission protocols, and interference control mechanisms might be incorporated into the model. Together, these design components provide a system that is intrinsically more energy-efficient than the alternative paradigm. In conclusion, the sophisticated optimization methodologies, environmental flexibility, and efficient overall design of the suggested methodology are significantly responsible for its better energy efficiency in the context of gearbox power variations. These components guarantee that the system maintains the performance requirements for dependable communication in crucial healthcare situations in addition to helping to lower energy usage.

Although the results reported in this study are purely based on simulated data created with MATLAB, the simulation scenarios were carefully crafted as closely resemble real-world situations as possible. We included realistic factors such as (transmission power, pathloss, SINR, etc.) based on values documented in contemporary literature and standards. These simulations allowed us to evaluate the proposed system’s robustness and performance under controlled yet practical situations. However, it is vital to recognize that real-world deployments can provide unexpected problems, such as hardware constraints, environmental variability, and unpredictable user behavior. As a result, while our findings illustrate the theoretical feasibility and performance potential of the proposed model, more validation through physical implementation and field testing is required to properly assess its usefulness in real-world circumstances. We intend to implement and evaluate the model in a real-time environment using (e.g., actual IoT devices, vehicle communication modules, or other relevant hardware), which will help capture additional factors such as latency variability, power consumption under real-world workloads, and system scalability. This stage is crucial for ensuring the model’s applicability and dependability beyond the simulated environment.

## 5 Conclusion

In this paper, both proposed model whether analytical or deep learning models were used to offer a novel communication strategy for effective interaction between chronic patient IoT devices and emergency vehicles. To guarantee that the data sent to emergency vehicles is correct and dependable and to prevent any interference from any other devices sharing the same the IoT transmitter device the same spectrum, the problem of optimizing energy efficiency and achievable data rate has been considered. This interference could affect the quality of the data received, which would subsequently affect the data sent to the emergency vehicle. Because of the interrupted received data, the emergency vehicle was unable to make an appropriate decision, endangering the patient’s life. Finding the optimal required distance between the emergency vehicle and the chronic patient IoT is the first step in addressing the difficulty of optimizing energy economy and the achievable data rate which was addressed using Lagrange optimization technique.MATLAB is then used to simulate this distance. As a result, a model recommendation was made for the subsequent 1D-CNN-based deep learning model. 1D-CNN is ideal for real-time applications since it is designed to have the least amount of computational complexity possible, allowing for processing energy efficiency and an overall achievable data rate. As a result, the deep learning model used for chronic patient IoT was able to determine the ideal transmission distance to achieve a nearly optimal result. Therefore,utilizing both approaches, the optimal transmission distance between p-IoT and the emergency vehicle has been estimated. To satisfy the required system performance criteria, the analytics of the results of anticipating the optimal necessary gearbox distance between p-IoT and emergency vehicle were presented. Based on the outcomes achieved in terms of the two suggested metrics which are the energy efficiency and achievable data rate. Considering the results obtained in relation to the two recommended metrics which are energy efficiency and achievable data rate, it has been found that the proposed model may exhibit the hightest performance under different environmental situations. Moreover, it has been shown that the necessary transmission distance can vary based on multiple factors, such as the p-IoT transmission power (*P*_*I*_), interfere devices transmission power, and interfere devices transmission distance, using analytical and deep learning techniques. The findings indicate that increasing the interference device’s transmission distance at maximum p-IoT and interference device power increases the necessary transmission distance between the p-IoT and the emergency vehicle. This is because increasing p-IoT transmission power helps to mitigate any potential interference that the transmission data may encounter, which in turn increases the required distance for transmitting data. Eventually, it can be demonstrated from the findings that the suggested model can accomplish the necessary IoT performance for chronic patients communication with any emergency vehicle while maintaining a respectable degree of system dependability and efficiency.
